# Salvianolic acid A alleviates the renal damage in rats with chronic renal failure^1^

**DOI:** 10.1590/s0102-8650201900204

**Published:** 2019-02-28

**Authors:** Guangming Zhang, Guanghua Cui, Shuangxi Tong, Qingxian Cao

**Affiliations:** I Master, Department of Urology, Affiliated Hospital, Beihua University, China. Technical procedures, final approval.; II Bachelor, Department of Urology, Affiliated Hospital of Beihua University, China. Acquisition of data, statistics analysis, final approval.; III Master, Department of Urology, Affiliated Hospital, Beihua University, China. Manuscript preparation, final approval.; IV Master, Department of Urology, Affiliated Hospital, Beihua University, China. Design of the study, critical revision, final approval.

**Keywords:** Salvianolic acid A, Kidney Failure, Chronic, Oxidative Stress, Bone Morphogenetic Protein 7, Smad6 Protein, Rats

## Abstract

**Purpose:**

To investigate the protective effects of salvianolic acid A (SAA) on renal damage in rats with chronic renal failure (CRF).

**Methods:**

The five-sixth nephrectomy model of CRF was successfully established in group CRF (10 rats) and group CRF+SAA (10 rats). Ten rats were selected as sham-operated group (group S), in which only the capsules of both kidneys were removed. The rats in group CRF+SAA were intragastrically administrated with 10 mg/kg SAA for 8 weeks. The blood urine nitrogen (BUN), urine creatinine (Ucr), creatinine clearance rate (Ccr), and serum uperoxide dismutase (SOD) and malondialdehyde (MDA) were tested. The expressions of transforming growth factor-β1 (TGF-β1), bone morphogenetic protein 7 (BMP-7) and Smad6 protein in renal tissue were determined.

**Results:**

After treatment, compared with group CRF, in group CRF+SAA the BUN, Scr, serum MDA and kidney/body weight ratio were decreased, the Ccr and serum SOD were increased, the TGF-β1 protein expression level in renal tissue was decreased, and the BMP-7 and Smad6 protein levels were increased (all P < 0.05).

**Conclusion:**

SAA can alleviate the renal damage in CRF rats through anti-oxidant stress, down-regulation of TGF-β1 signaling pathway and up-regulation of BMP-7/Smad6 signaling pathway.

## Introduction

 Chronic renal failure (CRF) is a kind of clinical disease in which the basic renal function cannot be maintained due to various causes of renal parenchymal damage and atrophy. The main clinical symptoms of CRF are the retention of metabolites, imbalance of water and electrolyte, acid-base imbalance and systemic involvement. CRF is the ultimate stage of the development of various chronic kidney diseases^1^. Renal interstitial fibrosis is a common pathological change of many chronic kidney diseases which eventually evolve into CRF^2^. The main manifestation of renal interstitial fibrosis is the high expression of transforming growth factor-β1 (TGF-β1)^3^. Smad protein is the key factors in the transduction of TGF-β1 family signal from receptor to nucleus^4^. Bone morphogenetic protein 7 (BMP-7) is an important anti-renal interstitial fibrosis factor influencing the signal transduction of TGF-β1/Smads pathway and reversing with TGF-β1^5^. Salvianolic acid A (SAA) is a water-soluble phenolic acid compound in the dried roots and rhizomes of *Salvia miltiorrhiza Bunge*. Pharmacological studies have confirmed that, SAA has significant anti-oxidative^6^, myocardial ischemia protective^7^, anti-thrombosis^8^, and anti-hepatic fibrosis effect^9^. *Salvia miltiorrhiza Bunge* has been widely used in the treatment of chronic kidney disease, and its anti-fibrosis effect has been recognized by the majority of physicians^10^. Zhang *et al.* ^11^ have reported that, SAA can attenuates kidney injury in 5/6Nx rats, which is attributed to its anti-inflammatory activities through inhibition of the activation of the NF-κB and p38 MAPK signaling pathways. In Li *et al.* ^12^, SAA may protect the renal function and improve the tubular function and renal pathology in rats with unilateral ureteral obstruction, which may be related to a reduction in inflammatory cytokines CCL5 and CXCL10 secretion. Are there any other mechanisms? In the present study, we observed the protective effect of SAA on the renal damage in rats with CRF and further discussed the underlying mechanisms, for providing an experimental basis for the clinical application of SAA to treatment of CRF.

## Methods

 This study was approved by the Animals Research Ethics Committee of the Affiliated Hospital of Beihua University. All animal procedures were in accordance with the Guide for the Care and Use of Laboratory Animals by the National Institutes of Health.

###  CRF modeling and animal grouping 

 The five-sixth nephrectomy model of CRF was established by ablation/infarction (A/I) method. The rats were anesthetized by intraperitoneal injection of 2% sodium pentobarbital (2 ml/kg). No tail response to clamping by hemostatic forceps represented the successful anesthesia. The rats were fixed in the right supine position. After shaving disinfection, a 2 cm-long skin incision perpendicular to the left side of the spine was made. Then, the muscles were cut and the left kidney was gradually exposed out of the body surface. After separating the renal capsule, 2/3 branches of left renal artery were ligated (single ligation of posterior and anterior descending branches). The operations were performed under the dissecting microscope. The injured kidney tissue surface was immediately compressed with gelatin sponge to stop bleeding until no obvious bleeding was visible to the naked eye. The kidney was placed in the abdominal cavity, and then the muscles and skin were sutured, followed by disinfection of surgical incision. The intraperitoneal injection of penicillin was performed once a day for 3 days to prevent the infection and death. After one week, the second surgery was performed with the method the same with before. After pulling out the body surface, the whole right kidney was removed by direct surgical excision. Then, the muscles and skin were sutured, followed by disinfection of surgical incision. The intraperitoneal injection of penicillin was performed once a day for 3 days. The biochemical indexes of rats were measured 4 weeks later. After eliminating the dead rats (4 rats died due to infection), the remaining 20 rats were randomly divided into model group (group CRF) and SAA treatment group (group CRF+SAA), with 10 rats in each group. Ten SD rats (male; 180-220 g) were randomly selected as sham-operated group (group S). In group S, the anesthesia and surgery method, procedure and duration were the same as those of modeling rats, but only the capsules of both kidneys were removed. No rat died in group S.

###  Treatment of animals 

 After one week, the rats in group CRF+SAA received the treatment with SAA by intragastric administration. The dose of SAA was 10 mg/kg (based on the pre-experiments). The administration was performed once per day, for 8 weeks. In the group S and group CRF, the same amount of normal saline substituting drug was intragastrically administrated.

###  Sample collection 

 After 8 weeks of treatment, the rats were weighed. The 24 h-urine volume was determined, and the 5 ml urine sample was preserved for index detection. The rats were anesthetized using 10 mg/L sodium pentobarbital, and the eyeball blood was taken. After centrifuging at 2000 r/min for 10 min, the serum was collected, and frozen in the refrigerator at -80^o^C for index detection. The median abdominal incision was made, and the left kidney was taken and preserved for index detection.

###  Determination of serum indexes 

 The renal function indexes including blood urine nitrogen (BUN), serum creatinine (Scr) and urine creatinine (Ucr) were determined using automatic biochemical analyzer. The creatinine clearance rate (Ccr) was calculated as follows: Ccr (ml/min) = [Ucr × 24 h-urine volume (ml) / [Scr × 1440 min]. The superoxide dismutase (SOD) level was determined by colorimetry. The malondialdehyde (MDA) level was determined by the barbiturate thiosulfate method. The operations were according to the instruction of kits (Sigma-Aldrich Corp., MO, USA).

###  Determination of TGF-β1, BMP-7 and Smad6 protein expressions in kidney tissues 

 The expression levels of TGF-β1, BMP-7 and Smad6 protein in kidney tissues were determined by western blotting assay. The kidney tissues were homogenized in a homogenizer. The protein was extracted using RIPA lysis buffer. The protein concentration was determined by Bradford method. The 10% SDS-PAGE was performed, and then the separated protein was transferred to the PVDF membrane. After blocking using l% BSA for 1h, the membranes were incubated with primary antibody (anti-TGF-β1, anti-BMP-7 and anti-Smad6) overnight at 4^o^C, followed by washing with PBS. The horseradish peroxidase-labeled second antibody was added, followed by incubation at room temperature for 1h. Visualization was accomplished by the enhanced chemiluminescence. The intensity of bands was calculated with Image J analysis software. The primary antibodies and secondary antibodies were provided by Beijing Dingguo ChangSheng Biotechnology Company (Beijing, China). The expression levels of TGF-β1, BMP-7 and Smad6 protein were presented by the ratio of target protein to β-actin (internal reference).

###  Statistical analysis 

 The statistical analysis was performed using SPSS 20.0 software (SPSS Inc., IL, USA). The data were presented as mean±SD. The difference between two groups was analyzed using one-way ANOVA with Bonferroni post-hoc test. P<0.05 presented statistically significant.

## Results

###  BUN, Scr and Ccr levels of rats after modeling 

 After CRF modeling, when compared with group S, in groups CRF and CRF+SAA the levels of BUN and Scr of rats were significantly increased, respectively (P < 0.05), and the Ccr was significantly decreased (P < 0.01). There was no significant difference of each index between groups CRF and CRF+SAA (P > 0.05) (Table 1).

**Table 1:** BUN, Scr and Ccr levels of rats after modeling.

Group	n	BUN (mmol/ml)	Scr (μmol/ml)	Ccr (ml/min)
S	10	5.76±1.32	29.93±3.27	1.54±0.36
CRF	10	17.78±2.98^*^	62.56±8.32^*^	0.66±0.16^*^
CRF+SAA	10	18.26±2.78^*^	62.83±10.55^*^	0.64±0.11^*^

^*^ *P* < 0.05 *vs.* group S. BUN, blood urine nitrogen; Scr, serum creatinine; Ccr, creatinine clearance rate.

###  Body weight of rats after treatment 

**Figure 1:**
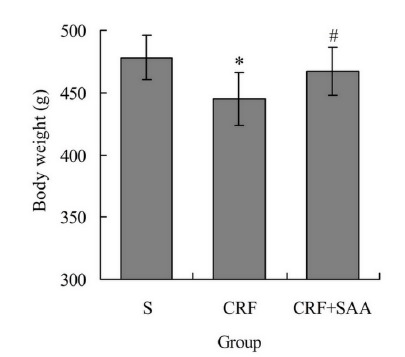
Body weight of rats after treatment. ^*^ *P* < 0.05 *vs.* group S; ^#^ *P* < 0.05 *vs.* group CRF.

 After treatment, the body weight of rats in groups CRF and CRF+SAA were 445.37±21.42g and 467.82±19.13g, respectively, obviously lower than 478.26±18.61g in group S, respectively (P < 0.05). In addition, the body weight in group CRF+SAA was obviously higher than that in group CRF (P < 0.05) (Fig. 1). 

###  BUN, Scr and Ccr levels of rats after treatment 

 After treatment, the levels of BUN and Scr of rats in groups CRF and CRF+SAA were remarkably higher than those in group S, respectively (P < 0.05), and the level of Ccr in groups CRF and CRF+SAA was remarkably lower than that in group S, respectively (P < 0.05). However, the levels of BUN and Scr of rats in group CRF+SAA were remarkably lower than those in group CRF, respectively (P < 0.05), and the level of Ccr in group CRF+SAA was remarkably higher than that in group CRF (P < 0.05) (Table 2).

**Table 2:** BUN, Scr and Ccr levels of rats after treatment.

Group	n	BUN (mmol/L)	Scr (μmol/ml)	Ccr (ml/min)
S	10	5.71±1.17	30.04±3.02	1.61±0.28
CRF	10	17.56±3.26^*^	63.52±7.68^*^	0.62±0.14^*^
CRF+SAA	10	12.26±2.46^*#^	55.83±7.15^*#^	0.83±0.15^*#^

* *P* < 0.05 *vs.* group S; ^#^ *P* < 0.05 *vs.* group CRF; BUN, blood urine nitrogen; Scr, serum creatinine; Ccr, creatinine clearance rate.

###  Serum SOD and MDA levels of rats after treatment 

**Figure 2:**
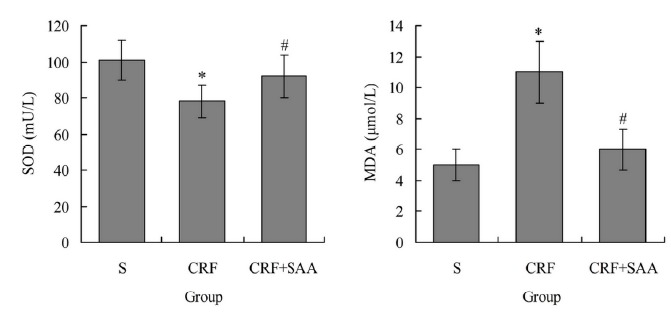
Serum SOD and MDA levels of rats after treatment. ^*^ *P* < 0.05 *vs.* group S; ^#^ *P* < 0.05 *vs.* group CRF. SOD, superoxide dismutase; MDA, malondialdehyde.

Figure 2 showed that, after treatment in group CRF the serum SOD level was 78.56±9.44 mU/L, significantly lower than 101.29±11.05 mU/L in group S (P < 0.05), and the serum MDA level was 11.21±2.19 μmol/L, significantly higher than 5.38±1.22 mU/L in group S (P < 0.05). The serum SOD level in group CRF+SAA was 92.82±12.11 mU/L, significantly higher than that in group CRF (P < 0.05), and the serum MDA level in group CRF+SAA was 6.18±1.31 μmol/L, significantly lower than that in group CRF (P < 0.05). There was no significant difference of each index between groups S and CRF+SAA (P > 0.05). 

###  Renal tissue TGF-β1, BMP-7 and Smad6 protein expression after treatment 

 After treatment, the renal tissue TGF-β1 protein expression level in groups CRF and CRF+SAA was significantly higher than that in group S, respectively (P < 0.05), and the BMP-7 protein level in group CRF and Smad6 protein level in groups CRF and CRF+SAA were significantly lower than those in group S, respectively (P < 0.05). When comparing with group CRF, in group CRF+SAA the TGF-β1 protein level was significantly decreased (P < 0.05), and the BMP-7 and Smad6 protein levels were significantly increased, respectively (P < 0.05). There was no significant difference of BMP-7 protein level between groups S and CRF+SAA (P > 0.05) (Table 3). 

**Table 3:** Renal tissue TGF-β1, BMP-7 and Smad6 protein expression after treatment (ratio to β-actin).

Group	n	TGF-β	BMP-7	Smad6
S	10	1.21±0.15	2.33±0.32	1.92±0.21
CRF	10	2.53±0.19^*^	0.91±0.14^*^	0.94±0.13^*^
CRF+SAA	10	1.66±0.21^*#^	2.11±0.22^#^	1.72±0.16^*#^

* *P* < 0.05 *vs.* group S; ^#^ *P* < 0.05 *vs.* group CRF. TGF-β1, transforming growth factor-β1; BMP-7, bone morphogenetic protein 7.

## Discussion

 In the present study, the CRF model of rats was established, and the effect of SAA on the renal damage in CRF rats were investigated. The result showed that, after CRF modeling, compared with group S, in groups CRF the body weight of rats was increased, the BUN and Scr levels were increased, and the Ccr level was decreased. This suggests that, there is obvious renal damage in CRF rats. After treatment, compared with group CRF, in group CRF+SAA the body weight was increased, the Scr and BUN levels were decreased, and the Ccr level was increased. This suggests that, SAA can alleviate the renal damage in CRF rats.

 Studies^13^ ^,^ ^14^ have shown that, there is obvious oxidative stress in CRF patients, and the oxidative stress can aggravate the condition and complications of CRF. Morena *et al.* ^15^ find that, the reactive oxygen species (ROS) play a key role in the pathophysiological process of kidney diseases. In oxidative stress state, the level of ROS increases and the activity of antioxidant enzymes decreases, leading to the destruction of dynamic balance between oxidation and antioxidation, which mediates the cell damage. In addition, Hirata *et al.* ^16^ have confirmed that, the oxidative stress is an important factor for aggravating the progression of CRF and occurrence of complications. Therefore, the anti-oxidation therapy is needed for CRF patient. Removal of ROS can reduce or prevent the oxidative stress, thereby reducing or inhibiting the resulting kidney damage. SOD is an important biological enzyme that scavenges ROS in body. It can blockade the lipid peroxidation, thus playing an antioxidant role^17^. MDA is one of products of lipid peroxidation and lipid metabolism^18^. Results of the present study showed that, there is obvious oxidative stress in CRF rats, which is related to the results of clinical study which shows that the oxidative stress exists in CRF patients^19^. The serum SOD level in group CRF+SAA was higher than group CRF, and the serum MDA level in group CRF+SAA was lower than group CRF. This indicates that, SAA can alleviate the oxidative stress, thus protecting to kidney tissues from damage by CRF. 

 Renal interstitial fibrosis is a common pathological change of many chronic kidney diseases which eventually evolve into CRF. It is an important index for predicting the condition of kidney diseases and has a significant impact on the prognosis. The anti-renal interstitial fibrosis has great significance in the treatment of CRF^20^. Cytokines play an important role in the process of renal interstitial fibrosis. They combine with the receptors on target cells and transmit the orders to the target genes through the respective signal transduction pathways, thus exerting the biological effects. TGF-β1 is a classical fibrogenic factor, and is a maker of renal fibrosis. Studies have shown that, TGF-β1 can reduce the degradation of extracellular matrix and lead to renal interstitial fibrosis and glomerulosclerosis. Effectively cutting off the signal transduction of TGF-β1 plays an important role in terminating and reducing the renal fibrosis^21^ ^,^ ^22^. In this study, after treatment, the renal tissue TGF-β1 protein expression level in group CRF was higher than group S, and that in group CRF+SAA was lower than group CRF. This suggests that, SAA can decrease the TGF-β1 expression in renal tissue, which may be related to its protective effect. 

 Smad protein is an important member in the downstream transducer of TGF-β1 signaling pathway. It is the substrate of TGF-β1 receptor kinase. Smad6 is an inhibitory type Smad, and is a negative regulator of TGF-β1. Smad6 cannot be phosphorylated by type I receptor and can inhibit the signal transduction^23^. BMP-7 is an important cytokine that inhibits the renal fibrosis. BMP-7 and TGF-β1 regulate each other through similar downstream Smad signaling pathway, and maintain the balance of biological activities^24^. It is found that, BMP-7 can increase the expression of Smad6, and inhibit the signal transduction of TGF-β1 and transcription of target genes^25^. The protection of renal function by BMP-7 is closely related to the up-regulation of Smad6 expression^26^. The results of this study showed that, after treatment, the renal tissue BMP-7 and Smad6 protein expression levels in group CRF were lower than group S, and those in group CRF+SAA were higher than group CRF. This indicates that, SAA can up-regulate BMP-7 and Smad6 expressions in renal tissue, which may be one of the important mechanisms of anti-renal fibrosis. 

## Conclusions

 Salvianolic acid A can alleviate the renal damage in CRF rats. The mechanism may be related to the anti-oxidative stress, down-regulation of TGF-β1 expression and up-regulation of BMP-7 and Smad6 expressions in renal tissue.
